# Insertion sequences related to ISAjo2 target p*dif* and *dif* sites and belong to a new IS family, the IS*1202* family

**DOI:** 10.1099/mgen.0.000953

**Published:** 2023-03-07

**Authors:** Christopher J. Harmer, Carol H. Pong, Ruth M. Hall

**Affiliations:** ^1^​ School of Life and Environmental Sciences, The University of Sydney, NSW 2006, Australia

**Keywords:** *Acinetobacter*, antibiotic resistance, *dif *modules, mobile genetic elements, p*dif*

## Abstract

Several insertion sequences (IS) found in various *Acinetobacter* species exhibit target specificity. They are found, in the same orientation, 5 bp from the XerC binding site of the p*dif* sites associated with *dif* modules in *Acinetobacter* plasmids, and searches revealed they are also found near chromosomal *dif* sites of *Acinetobacter* species. These IS are 1.5 kb long, bounded by 24–26 bp imperfect terminal inverted repeats (TIRs) and encode a large transposase of 441–457 aa. They generate 5 bp target site duplications (TSDs). Structural predictions of the ISAjo2 transposase, TnpAjo2, modelled on TnsB of Tn*7* revealed two N-terminal HTH domains followed by an RNaseH fold (DDE domain), a β barrel and a C-terminal domain. Similar to Tn*7*, the outer IS ends are 5′-TGT and ACA-3′, and an additional Tnp binding site, corresponding to the internal portion of the IR, is found near each end. However, the *Acinetobacter* IS do not encode further proteins related to those required by Tn*7* for targeted transposition, and the transposase may interact directly with XerC bound to a *dif*-like site. We propose that these IS, currently in the IS*1202* group in the not characterized yet (NCY) category in ISFinder, are part of a distinct IS*1202* family. Other IS listed as in the IS*1202* group encode transposases related to TnpAjo2 (25–56 % amino acid identity) and have similar TIRs but fall into three groups based on the TSD length (3–5, >15, 0 bp). Those with 3–5 bp TSDs may also target *dif*-like sites but targets were not found for the other groups.

## Data Statement

The DNA sequences of all insertion sequences (IS) utilized in this analysis are publicly available in the ISFinder database (https://isfinder.biotoul.fr), together with sequences of the encoded transposases.

## Introduction

**Fig. 1.**
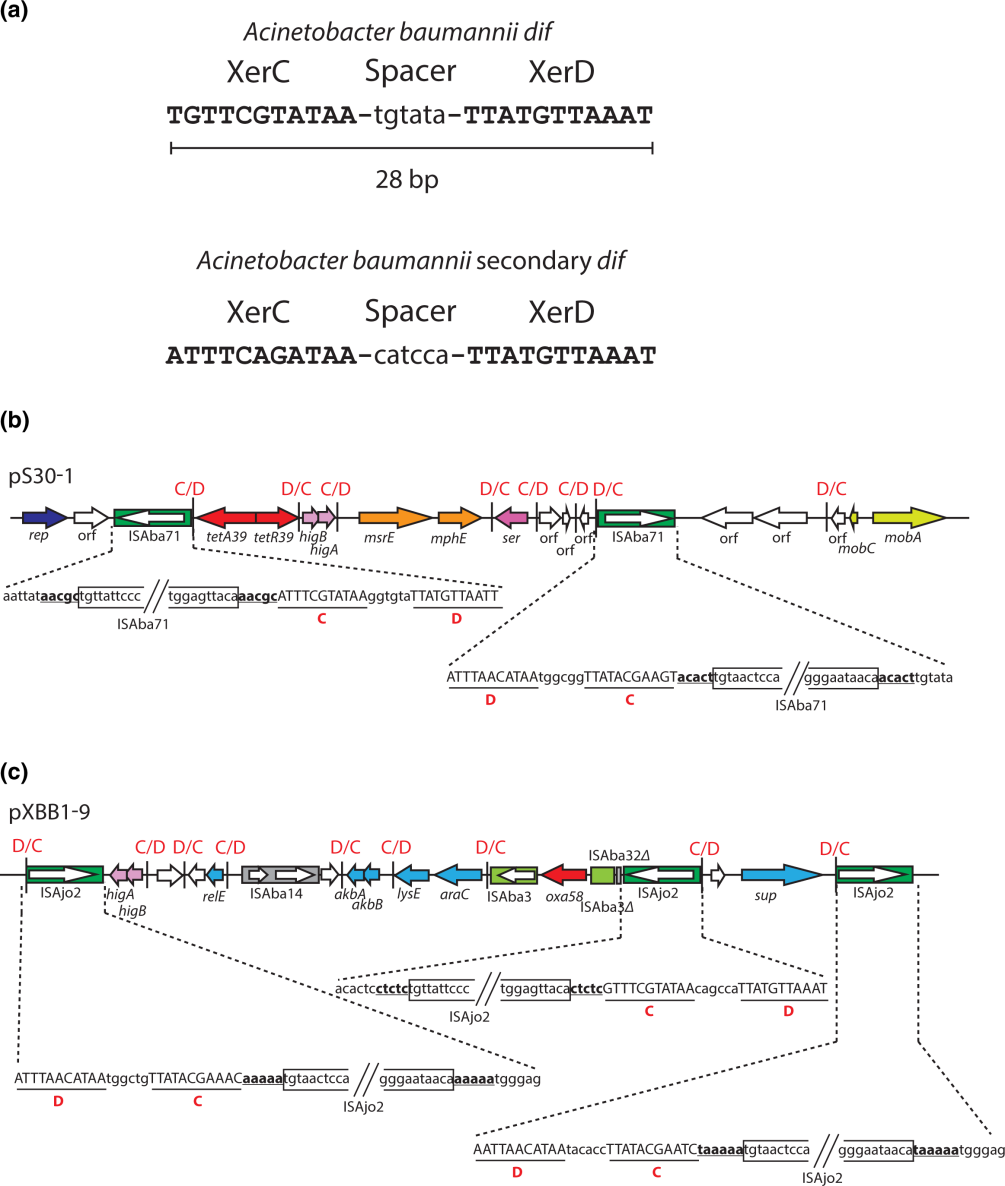
Chromosomal *dif* sites and insertion sequences (IS) associated with p*dif* sites. (**a**) *Acinetobacter baumannii* chromosomal *dif* and secondary *dif* sites. Drawn from strain A1 (GenBank accession number CP010781). The XerC and XerD binding sites are shown in capital letters and the 6 bp spacer is shown in lower case letters. Association of ISAba71 (**b**) and ISAjo2 (**c**) with p*dif* sites in plasmid pS30-1 (GenBank accession number KY617771) and pXBB1-9 (GenBank accession number CP010351), respectively. Arrows indicate the extents and orientations of ORFs, and genes with known function are labelled. IS are shown as boxes with an arrow indicating the extent and orientation of their transposase gene. Vertical bars represent p*dif* sites, with their orientation (C/D or D/C) shown above. p*dif* sites associated with ISAba71 or ISAjo2 are shown in detail below, with the XerC and XerD binding site capitalized and underlined, and the target site duplication shown as bold underlined text. The sequence at the left and right ends of each IS is boxed.

In recent years, many *Acinetobacter* plasmids have been reported to include a novel gene mobility system involving potentially mobile DNA units or modules containing one or more genes surrounded by p*dif* sites that resemble the chromosomal *dif* site [1–5]. The *dif* site is the recombination site in the terminal region of the chromosome that consists of two 11 bp sites, one recognized by XerC and one recognized by XerD, in inverse orientation and separated by a 6 bp spacer (Fig. 1a). The *dif* site is recognized and acted on by the XerC and XerD site-specific recombinases to resolve chromosomal dimers [6, 7]. The mobile units in the *Acinetobacter* plasmids were subsequently designated *dif* modules and the sites were designated p*dif* (plasmid *dif*) sites to distinguish them from the chromosomal *dif* site [8].

Insertion sequences (IS) and transposons (Tn) are often found in *dif* modules [8, 9, 10]. However, during an analysis of the *dif* modules in plasmid pS30-1 from a Global Clone 2 (GC2) *A. baumannii* from Singapore General Hospital, it was noted that a particular IS called ISAjo2-1 appeared to be closely associated with the p*dif* sites (Fig. 1b) [8]. Here, ISAjo2-1 has been re-named ISAba71, as the IS meets the ISFinder criteria for assignment of a separate name [11]. ISAba71 is a 1482 bp insertion sequence with 19/24 bp terminal inverted repeats (TIRs) and a *tnpA* gene that encodes a large 441 aa transposase [8]. The two copies of ISAba71 in pS30-1 were both located close (5 bp) to the XerC binding site of a p*dif* site and were surrounded by a 5 bp duplication, indicating that they generated a 5 bp target site duplication (TSD) upon insertion. Both copies of ISAba71 were in the same orientation with respect to the orientation of the p*dif* site (Fig. 1b).

ISAba71 shares 94.1 % DNA identity with ISAjo2 and the two transposases share 97.0 % amino acid identity and, when the sequence of the plasmid containing ISAjo2 (GenBank accession number CP010351 [12]) was re-examined [8], it was found that the two copies of ISAjo2 reported in this plasmid were also in the same orientation 5 or 6 bp away from the C side of p*dif* sites (Fig. 1c). Subsequently, ISAba32 [3] (1482 bp, 26 bp TIR, 5 bp TSD) which shares 73 % nucleotide identity with ISAjo2 and encodes a transposase that shares 72 % amino acid identity with the ISAjo2 transposase, was found to be located 5 bp away from the XerC side of a p*dif* site in the *Acinetobacter baumannii* plasmid pD36-4 (GenBank accession number CP012956). Recently, two additional *Acinetobacter* IS in this group (ISApi2 and ISAso2) have also been reported to be associated with p*dif* sites [13].

In the ISFinder database (www-is.biotoul.fr), ISAba71, ISAjo2 and ISAba32 and further relatives are currently assigned to the ISNCY (not characterized yet) category under the IS*1202* group. Here, we first examined the properties of the *Acinetobacter *-associated IS in the IS*1202* group and then compared them to other group members. We propose that these IS form a novel IS family, the IS*1202* family, that includes two main sub-groups, the IS*1202* sub-group and the ISAjo2 sub-group, distinguished by the length of the TSD they generate.

## Methods

### Databases and curation

To facilitate an accurate analysis of IS related to ISAjo2, a stand-alone database was built by first querying the ISFinder database (www-is.biotoul.fr) with the nucleotide and amino acid sequences of ISAjo2 and ISAba32 to identify all related IS in the IS*1202* group (last accessed 18 October 2022). The nucleotide and protein sequences for the ISFinder entries were downloaded and compiled into a custom blast database using Geneious version 7.1.9 (Biomatters).

A custom *dif* module database was constructed by searching the literature and collating previously identified and named *dif* modules. The nucleotide sequences of the modules were compiled into a custom blast database using Geneious version 7.1.9.

### Phylogeny

A ClustalW alignment (BLOSUM62 matrix, gap open cost 10, gap extend cost 0.1) was performed to align the predicted protein sequences, and the pairwise identity matrix output was used to check for any pairs of IS that fell within the current ISFinder rules for defining isoforms, namely sharing >98 % amino acid similarity and/or >95 % nucleotide identity with any other IS in the database. Only one representative of isoforms was included in the analysis. Alignments were exported to Adobe Illustrator (Adobe).

A phylogenetic analysis of the transposases of all IS in the curated data set was performed by building an un-rooted neighbour-joining tree from the ClustalW alignment. A consensus tree was built by resampling the analysis 10 000 times.

### Distribution and detection of IS targeting

To determine the prevalence of each of the *Acinetobacter-*associated IS, the GenBank non-redundant database was queried (last accessed 18 October 2022) with the nucleotide sequence of each IS as given in Table 1, and the number of entries that contained one or more copies of an IS sharing >95 % or >98 % nucleotide identity to the query IS was recorded. The origin of each sequence (plasmid or chromosome) was also recorded.

**Table 1.** IS belonging to the IS*1202* group found in *Acinetobacter* species in ISFinder

		Length (bp)			Length (aa)	Identity (%)	
**IS**	**Species**	**IS**	TIR*	**TSD**	**Tnp**	**TnpAjo2**	**TnpAba32**
**Cluster 1**							
ISAjo2	* Acinetobacter johnsonii *	1482	19/24	5 (6)	441	100.0	71.3
ISAba71†	* Acinetobacter baumannii *	1482	20/24	5	441	97.0	72.0
ISAso2	* Acinetobacter soli *	1482	20/24	5	457	94.7	70.9
ISApi2	* Acinetobacter pittii *	1482	19/24	5	457	92.9	70.2
**Cluster 2**							
ISAba32	* Acinetobacter baumannii *	1482	20/26	5	444	71.3	100.0
ISAba54‡	* Acinetobacter baumannii *	1481	19/26	5	441	71.1	93.4
ISAlw22	* Acinetobacter lwoffii *	1482	20/26	5	444	71.5	97.0

*The number of identical bases at the left and right ends and the length of the TIR.

†ISAjo2-1 in Blackwell & Hall [8].

‡An isoform of ISAba54, ISAso1, is also listed in ISFinder. It was discovered contemporaneously and reported by Moran *et al*. [13].

To examine targeting, the surrounds of three independent examples of each IS were examined and 50 bp of sequence on the left side of the IS (adjacent to the left TIR) was compiled into a custom blast database. The flanking sequences were queried for the XerD p*dif* consensus sequence (TTATGTTAATT or TTATGTTAAAT). The sequence was then examined manually for an XerC site at the right distance from the XerD sequence. If a potential *dif*-like site or related sequence was found, the orientation and distance of the IS from the *dif*-like site was recorded.

### Protein modelling

Transposase secondary structures were predicted by submitting the amino acid sequences to the Jpred4 server (http://www.compbio.dundee.ac.uk/jpred) using default parameters [14]. The Jpred output was mapped onto the Geneious-generated alignments of the transposases to confirm conservation of the transposase secondary structure. To predict the tertiary structure, the TnpAjo2 sequence was submitted to the homology modelling server Phyre2 [15] and input into the artificial intelligence program AlphaFold2 (ColabFold notebook run on Google Colaboratory using MMseqs2 and default settings; accessed July 2022) [16, 17]. The sequence coverage, pLDDT (predicted local distance difference test) and PAE (predicted alignment error) maps were used to assess the AlphaFold2 generated models and predicted domain packing. The top-ranked AlphaFold model for TnpAjo2 is presented here. All protein structures were rendered using PyMOL Molecular Graphics System v2.4.1.

## Results

### Properties of the *Acinetobacter *-associated IS

In the ISFinder database, in addition to ISAjo2, there are currently six other IS listed as being of *Acinetobacter* origin in the ISNCY category of IS under the IS*1202* group (Table 1). Four of these IS, ISAba32, ISAba71, ISAso2 and ISApi2, have previously been reported to be located 5 bp away from the XerC binding site of a p*dif* site in *Acinetobacter* plasmids in a specific orientation and surrounded by a 5 bp TSD [3, 8, 13]. In addition, a further copy of ISAjo2, found here in a re-examination of the pXBB1-9 plasmid sequence (GenBank accession number CP010351), was also located 5 bp away from a p*dif* site (Fig. 1c). Another two IS, ISAlw22 and ISAba54, had been noted but their location was not examined [18, 19]. Here they were also found to be located 5 bp away from a p*dif* site. A seventh IS listed in ISFinder, ISAso1 [13], is an isoform of ISAba54 that was discovered contemporaneously to ISAba54 [18]. Hence, these IS all clearly target *dif*-like sites and they generate a 5 bp TSD.

Pairwise analysis of the IS revealed that the six IS shared 67.7–94.1% nucleotide identity to ISAjo2 (Table 2), and the encoded transposases share 71.5–97.1 % amino acid identity to the ISAjo2 transposase (Table 1 and Table S1, available in the online version of this article). Given that ISAba71, ISAba32, ISAba54, ISApi2, ISAlw22 and ISAso2 all share a close relationship with ISAjo2, they will be referred to as the ‘ISAjo2 type’ henceforth. Based on amino acid identity >90 %, they fall into two distinct clusters. Cluster 1 includes ISAjo2, ISAba71, ISAso2 and ISApi1, and the second cluster includes ISAba32, ISAba54 and ISAlw22.

**Table 2.** Nucleotide identity (%) between ISAjo2-type IS

	ISAjo2	ISAba71	ISAso2	ISApi2	ISAba32	ISAba54	ISAlw22
ISAjo2	100	94.06	93.52	89.61	68.09	67.73	67.61
ISAba71		100	94.06	90.15	69.51	68.61	69.17
ISAso2			100	90.22	68.36	67.59	68.15
ISApi2				100	67.55	67.19	67.68
ISAba32					100	89.33	93.99
ISAba54						100	89.4
ISAlw22							100

**Fig. 2.**
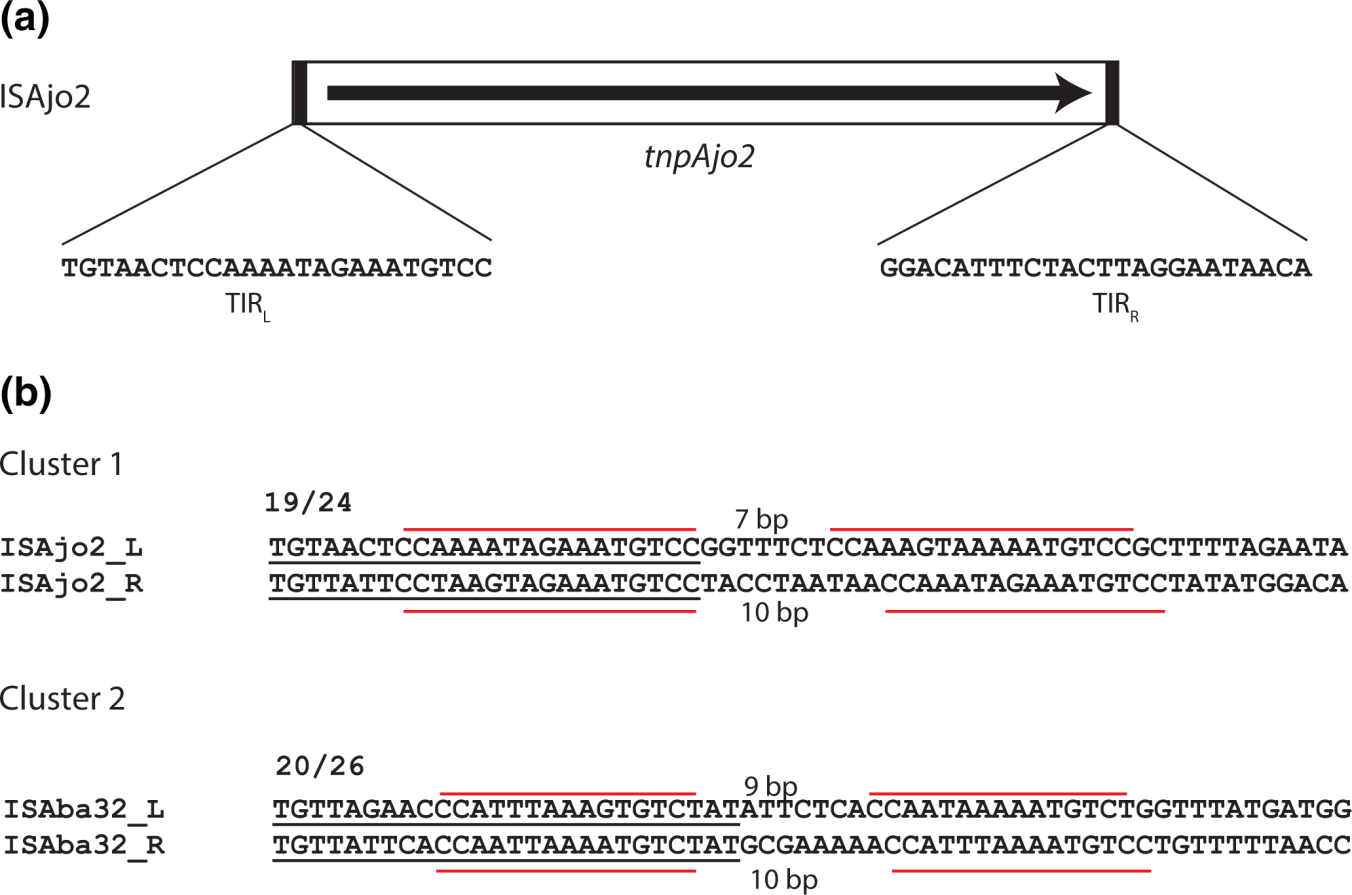
Structure of ISAjo2-type IS. (**a**) Schematic of ISAjo2, with the left (TIR_L_) and right (TIR_R_) inverted repeat sequences shown below. The arrow indicates the extent and orientation of the transposase gene. (**b**) Left and right ends of representative cluster 1 (ISAjo2) and cluster 2 (ISAba32) IS. TIRs are underlined, and repeated sequences are marked with a red line above the sequence. The spacing between the repeats is indicated above and below the sequence. Full cluster 1 and cluster 2 comparisons are shown in Fig. S1.

The ISAjo2 type IS are all 1481 or 1482 bp in length, and manual examination of the outer ends identified a 24–26 bp imperfect TIR with either 19 or 20 nn identical in their left and right ends (Table 1). The outermost bases are 5′-TGT and ACA-3′ (Fig. 2b), as found in the Tn*7* family [20, 21]. The Tn*7* TnsB transposase has binding sites that are found internal to the transposon [21] and, when the sequences of ISAjo2 and ISAba32 cluster members were examined, each IS possessed a second copy of the inner 19 bp of the TIR. The internal binding site was 7–10 bp from the TIR and the configuration differed slightly between the ends and between the two clusters (Figs 2b and S1).

### Predicted structure of the ISAjo2 transposase, TnpAjo2

**Fig. 3.**
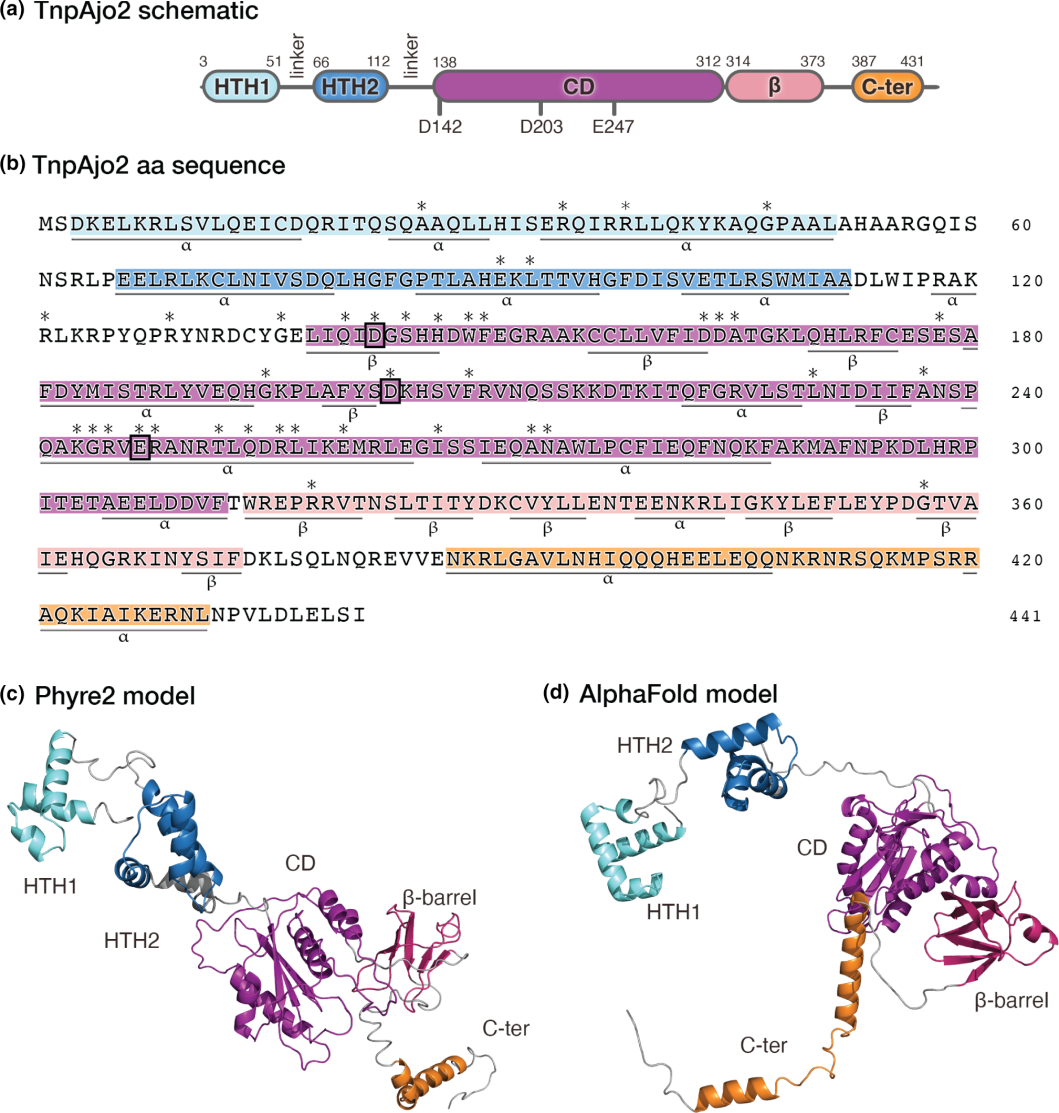
ISAjo2-encoded transposase TnpAjo2. (**a**) Schematic of TnpAjo2 with extent of the main protein domains and features identified represented as oblongs. The position of the catalytic residues (D142, D203 and E247) in the catalytic domain (CD) are indicated. (**b**) TnpAjo2 amino acid sequence with the α-helices and β-strands underlined as predicted by AlphaFold. The DDE catalytic residues are boxed. (**c**) Phyre2- and (**d**) AlphaFold-generated protein models of transposase TnpAjo2. For (a)–(d), the major domains and structures are labelled and the shared colour scheme is as follows: the N-terminal HTH1 and HTH2 helical bundles are coloured light and dark blue, respectively, and the DDE catalytic, β-barrel and C-ter are coloured magenta, pink and orange, respectively. An asterisk indicates amino acid residues that are either completely conserved or highly conserved (26 of 28 identical) in the alignment of IS*1202* transposases (Fig. S2).

A model of the TnpAjo2 protein was generated using the Phyre2 homology modeller, and 427 out of 441 aa (97 %) were modelled with >90 % confidence. TnpAjo2 modelled closely to the TnsB transposition protein encoded by Tn*7* (PDB: 7PIK; [22]) and the bacteriophage Mu transposase (PDB: 4FCY; [23]). TnpAjo2 was predicted to have two N-terminal helix–turn–helix (HTH) tri-helical bundles, the RNaseH fold or DDE catalytic domain immediately followed by a small β-barrel structure, and a bi-helical C-terminal domain (C-ter) (Fig. 3). The AlphaFold artificial intelligence modeller was also used for comparison, and the protein models produced by both Phyre2 and AlphaFold shared high structural similarity and domain packing (Fig. 3c).

### Prevalence and spread of ISAjo2 type IS

The IS in ISFinder are named after the species in which they were first observed, and though this can give the impression that they are confined to a particular bacterial species, that is not always so. To understand the importance that these ISAjo2 group p*dif* site hunters may play in the evolution of *Acinetobacter* plasmids, we sought to understand how prevalent each IS is and whether they have been found outside the genus *Acinetobacter *. For each of the IS in Table 1, the number of GenBank entries containing regions with 99 % coverage and a nucleotide identity >95 % or >98 % to the query sequence was recorded, along with the species associated with the GenBank entry (Table 3).

**Table 3.** Prevalence of *Acinetobacter* IS in the GenBank non-redundant database (accessed 18 October 2022)

	No. of GenBank entries		
IS	>98 % DNA identity (plasmid/chromosome)	>95 % DNA identity (plasmid/chromosome)	Species∗
**Cluster 1**			
ISAjo2	1 (1/0)	14 (9/5)	*A. baumannii, A. bereziniae, A. chinensis, A. defluvii, A. indicus, A. johnsonnii, A. towneri*
ISAba71	25 (15/10)	70 (42/28)	*A. baumannii, A. bereziniae, A. chinensis, A. defluvii, A. haemolyticus, A. indicus, A. johnsonnii, A. junii, A. lwoffii, A. nosocomialis, A. towneri, A. ursingii*
ISApi2	22 (15/7)	26 (18/8)	*A. baumannii, A. defluvii, A. haemolyticus, A. johnsonnii, A. nosocomialis, A. pittii, A. seifertii*
ISAso2	2 (2/0)	15 (10/5)	*A. baumannii, A. bereziniae, A. chinensis, A. defluvii, A. indicus, A. johnsonni, A. junii A. towneri*
**Cluster 2**			
ISAba32	4 (3/1)	31 (16/15)	*A. baumannii, A. johnsonni, A. lwoffii, A. radioresistens*
ISAba54	6 (2/4)	6 (2/4)	*A. baumannii, A. pittii*
ISAlw22	6 (2/4)	13 (6/7)	*A. johnsonnii, A. lwoffii, A. portensis, A. wuhouensis*

*Species in which IS sharing >95 % nucleotide identity to the query sequence are found.

The original ISAjo2 is not common. It does not match at >98 % identity to any other sequence in the GenBank non-redundant database, and only 14 GenBank entries (nine plasmids and five chromosomes) contain isoforms sharing >95 % nucleotide identity with it (Table 3). ISAba71 is the most prevalent member of this group of IS, with 70 GenBank entries containing one or more IS with >95 % identity, 25 of which share >98 % identity, to the ISAba71 sequence (Table 3). ISAba71 and its isoforms (>95 % identity) are found in the greatest number of *Acinetobacter* species, namely *A. baumannii, A. bereziniae, A. chinensis, A. defluvii, A. haemolyticus, A. indicus, A. johnsonnii, A. junii. A. lwoffii, A. nosocomialis, A. towneri* and *A. ursingii *. The 70 GenBank entries include 42 entries identified as complete plasmids, and 28 entries identified as circular chromosomes. The latter group are examined further below.

ISAba32 is the next most prevalent, with 31 instances (16 plasmid and 15 chromosomal GenBank entries) found in six different *Acinetobacter* species (Table 3). ISAba54, which was first reported only recently [18], is only found in a total of six GenBank entries and is currently confined to *A. baumannii* and *A. pittii *. For ISAlw22, ISAso2 and ISApi2, 13–26 instances of each IS were detected in a range of *Acinetobacter* species in both plasmids and chromosomes.

Hence, the IS examined were never found outside of the genus *Acinetobacter *. In addition, the number of instances of IS sharing >98 % nucleotide identity (often over 99 % identity) in multiple different *Acinetobacter* species is indicative of relatively recent spread.

### *Acinetobacter* IS of the ISAjo2 type are also p*dif* site hunters

To explore whether these IS had always targeted a p*dif* site in an *Acinetobacter* plasmid, the location of three independent examples of each IS in a plasmid were examined. As the p*dif* site is adjacent to the left TIR, as shown in Fig. 2(a), 50 bp of sequence on the left side of the IS was retrieved from the GenBank non-redundant database. The XerD binding site (TTATGTTAAAT or TTATGTTTAATT) is more highly conserved than the XerC sequence, so the adjacent sequence was manually examined for the XerD consensus to determine if the IS was associated with a p*dif* site. In each instance, the IS was found to be 5 bp away from the XerC side of a p*dif* site, always in the same orientation relative to the p*dif* site, and surrounded by a 5 bp TSD (Table 4). Identical spacer sequences were identified in several instances, indicating that the same p*dif* site had been targeted on independent occasions by different IS related to ISAjo2.

**Table 4.** Representative p*dif* sites targeted by IS of *Acinetobacter* origin

IS	Accession*	TSD	C†	Spacer	D†
ISAjo2‡	CP010351 (1)	AAAAA	GTTTCGTATAA	CAGCCA	TTATGTTAAAT
ISAjo2‡	CP010351 (2)	CTCTC	GTTTCGTATAA	CAGCCA	TTATGTTAAAT
ISAjo2‡	CP010351 (3)	TAAAAA	GATTCGTATAA	GGTGTA	TTATGTTAATT
ISAjo2-i‡	EF102240	ATCTC	GTTTCGTATAA	CAGCCA	TTATGTTAAAT
ISAjo2-i‡	CP078043	TTTTA	ATTTCGTATAA	GGTGCA	TTATGTTAATT
ISAba71§	KY617771 (1)	AACGC	ATTTCGTATAA	GGTGTA	TTATGTTAAAT
ISAba71§	KY617771 (2)	AGTGT	ACTTCGTATAA	CCGCCA	TTATGTTAAAT
ISAba71§	CP084298 (1)	TTGGG	AATTCGTATAA	CGTGTA	TTATGTTAATT
ISAba71§	CP084298 (2)	GTTCA	ACTTCGTATAA	CGTGTA	TTATGTTAATT
ISAba71§	CP084298 (3)	ACAGA	ATTTCGTATAA	CCACCA	TTATGTTAAAT
ISAba32	CP051211	TTTTG	GATTCGTATAA	GGTGTA	TTATGTTAATT
ISAba32	CP084302 (1)	AGTGT	ACTTCGTATAA	CCGCCA	TTATGTTAAAT
ISAba32	CP084302 (2)	AACAC	GTTTCGTATAA	GGTGTA	TTATGTTAATT
ISAba54	CP082952	ATCTC	GTTTCGCATAA	CAGCCA	TTATGTTAAAT
ISAba54	CP042365	CAGAG	ATTACGTATAA	CGTGTA	TTATGTTAATT
ISAba54	JX101647	CAAGC	ATTACGTATAA	CGTGTA	TTATGTTAATT
ISAso2	JX101647	CTCTC	GTTTCGTATAA	CAGCCA	TTATGTTAAAT
ISAso2	CP078043	ATCTC	GTTTCGCATAA	CAGCCA	TTATGTTAAAT
ISAso2	CP045127	AGTGT	ACTTCGTGTAA	TCGCCA	TTATGTTAAAT
ISApi2	CP033536	ATAGC	ATTTCGTATAA	GAGTTT	TTATGTTAAAT
ISApi2	CP051863	CTCTC	GTTTCGTATAA	CAGCCA	TTATGTTAAAT
ISApi2	MK134375	TTGGG	AATTCGTATAA	CGTGTA	TTATGTTAATT
ISAlw22	CP054822	AGGGC	CTTTCGTATAA	TGCCCA	TTATGTTAAAT
ISAlw22	CP032290 (1)	AACGC	ATTTCGTATAA	GGTGTA	TTATGTTAATT
ISAlw22	CP032290 (2)	TATAC	GTTTCGTATAA	GCTCTA	TTATGTTAAAT

*Multiple IS in a single GenBank entry are numbered in parentheses.

†Red lettering indicates bases that differ from the p*dif* consensus sequence.

‡ISAjo2 denotes the original sequence, and ISAjo2-i denotes an isoform sharing >95 % nucleotide identity.

§Five examples reported for ISAba71 due to multiple examples being found in two GenBank entries.

### The chromosomal *dif* site can also act as a target for ISAjo2 type IS

The IS of the ISAjo2 type that had been examined in any detail previously were all found in plasmids adjacent to p*dif* sites. The observation that several of the matches in the GenBank non-redundant database were chromosomal entries (Table 3) raised the possibility that these IS may be able to target the chromosomal *dif* site and/or the additional *dif*-like site that has been found in several *Acinetobacter* species [5]. Alternatively, a plasmid fragment containing a p*dif* site may have been integrated into the chromosome. To distinguish these possibilities, the chromosomal locations of the most prevalent member of the group, ISAba71, were examined. The eight chromosomal GenBank accessions with an IS that shared >99 % nucleotide identity with ISAba71 were queried with the ISAba71 sequence, and the sequence surrounding each hit was manually examined.

In six instances (Table 5), ISAba71 was found 5 bp away from the XerC binding site of the chromosomal *dif* site in *A. baumannii *, *A. haemolyticus, A. indicus* and two unidentified *Acinetobacter* spp. In *A. baumannii *, the chromosomal *dif* site (TGTTCGTATAA TGTATA TTATGTTAAAT, C–spacer–D) is located between locus tags ABA1_01921 and ABA_01922 in the A1 reference sequence (GenBank accession number CP010781 [24]). In *A. haemolyticus, A. indicus* and the unidentified *Acinetobacter* spp., the sequences of chromosomal *dif* sites vary (Table 5), but the sites are all found in a similar location relative to the terminal region of the chromosome. In one instance, a second copy of ISAba71 was found adjacent to one of the secondary chromosomal *dif* sites in an *A. indicus* sequence (GenBank accession number CP029254), in addition to a copy adjacent to the primary *dif* site (Table 5). Finding ISAba71 adjacent to the XerC binding site of the chromosomal *dif* site demonstrated for the first time that in addition to targeting p*dif* sites, ISAba71 is also able to target the chromosomal *dif* site.

**Table 5.** Chromosomally located *dif* sites targeted by ISAba71 or isoforms

				Sequence				
Species	Molecule	Accession	Location*	Left flank†	C	Spacer	D	Right flank
* A. baumannii *	Chromosome	CP018254	Chromosomal *dif*	actat**ataat**	TGTTCGTATAA	tgtata	TTATGTTAAAT	aaaagatttg
* A. baumannii *	Chromosome	CP091373	Chromosomal *dif*	actat**ataat**	TGTTCGCATAA	tgtata	TTATGTTAAAT	aaaggatttg
* A. haemolyticus *	Chromosome	CP031984	Chromosomal *dif*	atgtt**ggtaa**	ATTGCGCATAA	tgtata	TTATGTTAAAT	agaatatccg
* A. indicus *	Chromosome	CP094254	Chromosomal *dif*	ctata**gactg**	ATTTCGTATAA	tgtata	TTATGTTAAAC	gaagtgtact
* A. indicus *	Chromosome	CP094254	Secondary *dif*	ttaca**agtcg**	AATTCGCATAA	cagcca	TTATGTTAAAT	agaaattttt
* Acinetobacter* sp.	Chromosome	CP028561	Chromosomal *dif*	tgtta**taata**	ATTGCGTATAA	tgtata	TTATGTTAAAT	agaatatcta
* Acinetobacter* sp.	Chromosome	CP026616	Chromosomal *dif*	agatt**ttttg**	ACTTCGCATAA	ggtgta	TTATGTTAATT	gtaggaaaac
* A. indicus *	Chromosome	CP046392	p*dif* in chromosome	ataca**agtcg**	ACTTCGCATAA	cagcca	TTATGTTAAAT	ggaaggaagc
* A. indicus *	Chromosome	CP024620	p*dif* in chromosome	attca**agtgt**	ACTTCGTATAA	tatcca	TTATGTTAAAT	agagatggct

*Chromosomal *dif* is the site at the terminus.

†Bold sequence denotes 5 bp TSD.

Examination of the remaining chromosomal GenBank entries with an ISAba71 or isoforms sharing >99 % nucleotide identity revealed instances of plasmid fragments containing p*dif* sites incorporated into the chromosome. In two instances (GenBank accession numbers CP046392 and CP024620), ISAba50 (an IS*256* family member) had incorporated a fragment of an *Acinetobacter* plasmid into the chromosome of an *A. indicus* strain. These plasmid fragments each contained a single p*dif* site, and this p*dif* site had been targeted by ISAba71 (Table 5).

ISAba54, ISApi2 and ISAlw22 also produced chromosomal hits sharing >99 % identity with the respective IS nucleotide sequence. Examination of those GenBank entries revealed evidence for ISApi2 next to the chromosomal *dif* site (seven examples) in *A. baumannii, A. junii* and *A. haemolyticus *. ISAlw22 was next to a secondary chromosomal *dif* site (in four examples) in *A. lwoffii *. ISAba54 was found in the *A. baumannii* chromosome in four GenBank entries in an IS*1008*-bounded pseudo-compound transposon (PCT) and this PCT is the location where ISAba54 was originally found [18]. It includes multiple p*dif* sites, only one of which has been targeted by ISAba54.

### ISAba71 and isoforms are associated with many different *dif* modules

Over the past few years, several different *dif* modules have been reported, including those containing antibiotic resistance genes, toxin–antitoxin genes and other genes of unknown function [3, 5, 8]. However, the discoveries of these modules are often chance events arising during the analysis of plasmids or complex mosaic resistance regions. The association of IS of the ISAjo2 type with p*dif* sites presents a unique pathway to identifying novel *dif* modules within *Acinetobacter* species and potentially more broadly.

As a proof-of-concept, 13 plasmid sequences from the GenBank non-redundant database that included one or more IS with >99 % nucleotide identity to ISAba71 were examined in detail to determine the precise location of the IS. First, the p*dif* site that had acted as the target was identified and the sequence on the other side of the IS was examined for a second p*dif* site in the opposite orientation, giving the configuration of a C-type *dif* module. In total, ISAba71 was found associated with 12 different *dif* modules (Table 6). Each *dif* module was then compared to a custom database of 19 known *dif* modules to determine if it was a novel module. Several of the modules had been reported previously, including the *oxa58* module, the *ser* module, the *higAB* and *abkAB* toxin–antitoxin modules, and the *relE* module [1, 3, 5, 8, 10]. However, five *dif* modules were identified that to the best of our knowledge have not been reported previously (shaded in Table 6). This included two novel toxin–antitoxin modules (designated *higAB2* and *higAB3*) that each share approximately 94 % nucleotide identity with the *higAB* module described previously, and five modules containing genes of unknown function. In pS30-1, one copy of ISAba71 is in the plasmid backbone (Fig. 1b) adjacent to a p*dif* site. In two more examples, ISAba71 is also in a plasmid backbone adjacent to a p*dif* site. Using IS to identify p*dif* sites opens the possibility of discovering novel *dif* modules in the surrounding sequence.

**Table 6.** *dif* modules targeted by ISAba71 found in plasmids

Plasmid	Accession	Species	*Dif* module(s) targeted*
pS30-1	KY617771	* A. baumannii *	Plasmid backbone Hyp11
pLCH22-2	CP084298	* A. baumannii *	*ser oxa58* *higAB2*
pAJ_351–2	CP078019	* A. junii *	*higAB oxa58*
pOXA58_010030	CP029396	* A. defluvii *	*abkAB*
pNDM1_010045	CP028560	* Acinetobacter* sp.	*higAB oxa58*
p3_010045	CP039669	* Acinetobacter* sp.	Plasmid backbone
pAF-401	CP018255	* A. baumannii *	Hyp13 Hyp14 Hyp15
p_ajICE_NC	CP090417	* A. johnsonii *	*higAB3*
pXMC5×702	CP084302	* A. pseudolwoffii *	*relE*
pH7-250	CP072550	* A. lwoffii *	*higAB* Tox3
pBDT2044-7	CP094253	* A. variabilis *	*oxa58*
pMMCU2	GQ476987	* A. baumannii *	Plasmid backbone
pNDM-32	LN833432	* A. baumannii *	*msrE-mphE*

*Shading denotes a novel *dif* module that has not been reported previously. Hyp, *dif* module containing genes encoding proteins with no known function; Tox, *dif* module containing genes encoding toxin/antitoxin proteins.

### The IS*1202* group in ISNCY represents a new IS family

In addition to the seven IS of *Acinetobacter* origin, 21 other IS are listed in the IS*1202* group, ISNCY category of IS in ISFinder (Table 7). Among these IS recovered from ISFinder, 14 were identified in a Gram-negative bacterium, three in a Gram-positive bacterium and four in metagenomic sequence data. Eighteen of the IS range in size from 1327 to 1747 bp. Three larger IS, ISKpn21, ISKpn65 and ISRel10, are classified as transporter IS (tIS) as each includes an additional ORF. However, the reading frame in each of the three IS was different, and the encoded predicted proteins did not identify a protein of known function in the Pfam database.

**Table 7.** Other IS belonging to the IS*1202* family

		Length (bp)			Length (aa)	Identity (%)	
**IS**	**Species**	**IS**	**TIR**	TSD*	**Tnp**	**TnpAjo2**	**Tnp1202**
ISLad2	*Leclercia adacarboxylata*	1647	19/26	4	448	55.9	25.7
ISKpn65	* Klebsiella pneumoniae *	1980†	16/26	5	452	54.4	26.0
ISRel10	* Rhizobium etli *	2528†	15/25	5	473	46.1	24.6
ISRel26	* Rhizobium etli *	1634	17/24	5	457	46.1	24.3
ISMex6	* Methylobacterium extorquens *	1535	16/26	5	432	48.9	25.0
ISKpn21	* Klebsiella pneumoniae *	2278†	25/25	5	467	44.4	24.1
ISSen13	* Salmonella enterica *	1542	17/28	3	456	45.2	22.2
ISBcen27	* Burkholderia cenocepacia *	1690	16/26	5	496	45.5	24.5
IS*1202*	* Streptococcus pneumoniae *	1747	18/23	27	465	25.4	100.0
ISCARN63	Metagenomic data	1387	21/26	17	414	34.9	27.8
ISCARN62	Metagenomic data	1398	16/24	16	432	36.1	26.8
ISTde1	* Treponema denticola *	1327	18/27	17	394	34.7	27.8
ISCARN53	Metagenomic data	1404	19/26	16	407	35.0	28.1
ISShha1	*Shewanella halifaxens*	1737	18/26	16	507	28.5	28.1
ISHahy13	*Halanaerobium hydrogeniformans*	1497	19/24	17	447	28.2	34.4
ISVbsp4	* Vibrio* sp.	1737	19/24	16	504	29.8	27.1
ISVisp7	* Vibrio splendidus *	1742	16/24	16	506	25.6	27.9
ISSeq2	* Streptococcus equi *	1544	21/25	28	465	25.1	65.8
ISRor8	* Raoultella ornithinolytica *	1472	17/28	–	465	48.0	24.9
ISEsa1	* Enterobacter sakazakii *	1609	15/22	–	439	41.0	26.7
ISCARN112	Metagenomic data	1468	23/29	–	428	39.5	27.3

*A dash indicates no TSD detected when multiple examples were examined.

†The IS contains two ORFs, classified as a transporter IS (tIS).

Each IS encodes a predicted transposase ranging in size from 394 to 507 aa. However, the shortest, found in ISTde1, lacks the C-terminal domain (see Fig. S2) and ISTde1 is >60 bp shorter than the remaining IS. A pairwise analysis of the transposases revealed that they share 25–60 % amino acid identity with the ISAjo2 transposase. Identity to the IS*1202* transposase was less than 35 % except for ISSeq2 (65 %).

The TIR lengths recorded in ISFinder for the 21 IS range from 14 to 42 bp, but here a manual examination of each IS found that all TIRs were in the expected range (Table 7).

**Fig. 4.**
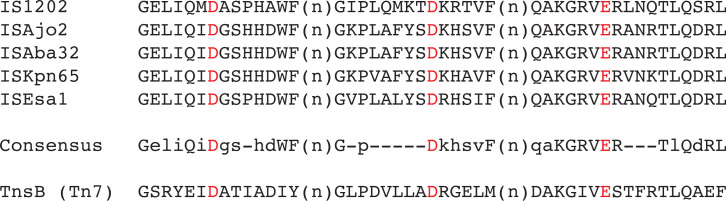
Conservation surrounding the DDE catalytic motif. One representative transposase from each group is included in the alignment. A capitalized letter in the consensus indicates the residue is conserved in at least 26/28 of the IS*1202* family transposases. A lower-case letter in the consensus indicates the residue is dominant at that position but is not completely conserved, and a hyphen indicates no dominant residue identified at that position. The DDE residues in the consensus are shown as red letters. The same regions from the Tn*7* TnsB transposase are shown below.

An alignment of the transposases encoded by the 28 IS*1202* group members (Fig. S2) revealed that most of the amino acids completely or highly conserved lie within the catalytic domain (marked with an asterisk in Fig. 3). In addition, characteristic motifs were found in the vicinity of the D and E residues. Notably, the amino acid found at the seventh position from the E was Q rather than the R or K found at this position in other DDE transposases [25, 26]. Here, we therefore propose that these IS form a distinct family and should be designated the IS*1202* family. These motifs were also compared to the corresponding region in the TnsB transposase of Tn*7* and the conserved motifs were shared (Fig. 4). However, the IS do not include additional proteins found in the Tn*7* transposon family.

### TSD lengths and transposase phylogeny separate IS in the IS*1202* family into subgroups

In some instances, a TSD length is not recorded in ISFinder, and the TSD data shown in Tables 1 and 7 were generated from a manual analysis of sequences containing each IS in the course of this study. The TSD lengths for the remaining IS in the current IS*1202* group (Table 7) indicate three sub-groups. One group includes IS with a short TSD of 3–5 bp and includes the ISAjo2 type (Table 1). Other subgroups are those with unusually long TSDs of 16–28 bp, and a few with no identifiable TSD. TSD length is one of the characteristics used to classify IS into families and into groups within families [11], and the distinct differences in TSD length point to potential mechanistic differences.

**Fig. 5.**
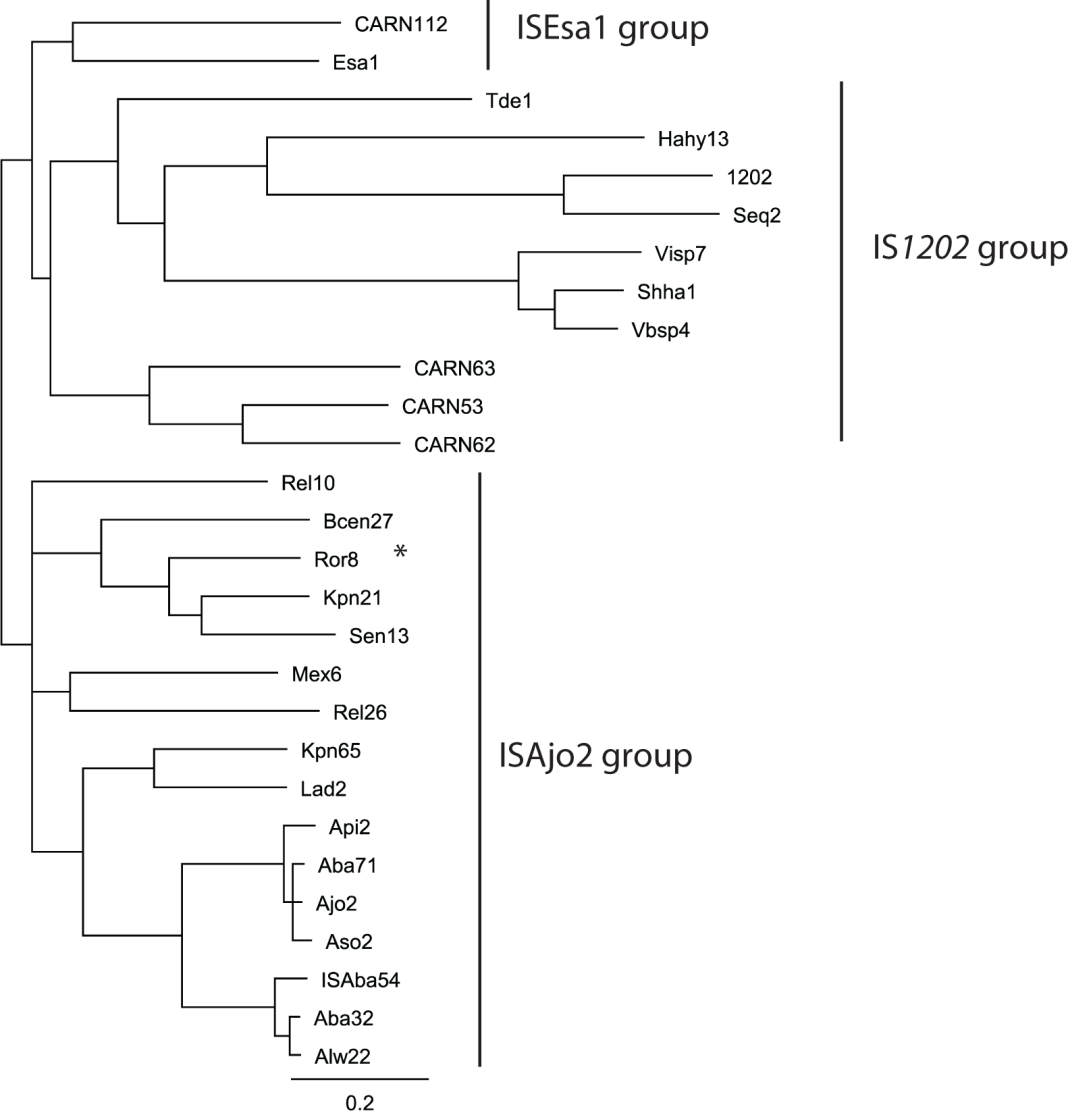
Un-rooted neighbour-joining pylogeny of IS*1202* family transposases. The consensus tree was reconstructed by resampling the analysis 10 000 times. IS belonging to the IS*1202* group and ISAjo2 group are marked with labelled vertical bars. An asterisk next to ISRor8 denotes clustering by phylogeny with the short TSD ISAjo2 group, despite no evidence of a TSD being generated in available sequence data. The scale bar indicates the consensus support %.

A phylogenetic analysis of the transposases of all 28 IS in the curated data set, performed by building an un-rooted neighbour-joining tree (Fig. 5), was largely consistent with the sub-groupings. Repetition of the analysis using the transposase of IS*903* from the IS*5* family as an outgroup produced a tree with the same topology (data not shown). The transposases fall broadly into three sub-groups, corresponding to the clusters determined by TSD length. One sub-group contained the transposases of ISAjo2 and the other IS found in *Acinetobacter* species as well as 13 close relatives with short TSDs, including those found in *Klebsiella pneumoniae *, *Salmonella enterica* and *Burkholderia cenocepacia *, amongst others. IS*1202* and the nine other IS with longer TSDs formed a distant sub-group, while two IS with no detectable TSD (ISEsa1 and ISCARN112) made up the third sub-group. The only exception to this grouping was ISRor8 which had no detectable TSD but clustered with the short TSD group in the transposase phylogeny and will require further examination.

### Do the further members of the IS*1202* family also target *dif*-like sites?

The strategy used to detect p*dif* targeting in the *Acinetobacter* ISAjo2 group IS was employed to examine three independent examples of each of the IS with 3–5 bp TSD (ISLad2, tISRel10, ISRel26, ISMex6, tISKpn21, tISKpn65, ISSen13, ISBcen27). The sequence adjacent to the left end of the IS was manually examined for the XerD consensus to determine if the IS was associated with a *dif*-like site. In each of the three examples examined, ISKpn21 and ISSen13 were found 5 or 3 bp away, respectively, from the C side of a conserved *dif/dif*-like site (Table 8). In one instance, ISKpn21 was found in a novel *dif* module containing a gene encoding a GNAT acetyltransferase in an HI1B plasmid (GenBank accession number CP079726). ISSen13 was found adjacent to a single *dif-*like site (found at bases 124327–124 354 in pSRC27-H, GenBank accession number CP058810) that is found in multiple sequences belonging to a widespread lineage (type 2) of HI1 plasmids [27, 28] that includes some of the earliest-recovered multiresistant HI1 plasmids. ISKpn65 was found in one instance 5 bp away from a site with a clear XerD binding site sequence, but the other two examples included several nucleotide changes in the XerD binding site sequence and very little conservation in the XerC site sequence (Table 8).

**Table 8.** Representative p*dif*-like sites targeted by other IS*1202* group IS

IS	Accession	TSD	C*	Spacer	D*
ISKpn21	CP024290	CCTTT	AGTGCGCATAA	TGTATA	TTATGTTAAAT
ISKpn21	CP079726	GCATT	AGTGCGCTTAA	TGTACG	TTATGTTAAAT
ISKpn21	CP075891	ATACA	GGTGCGCATAA	CAAAGA	TTATGTTAAAT
ISKpn21	MN661402	GACTT	AGTGCGCATAA	TGTACG	TTATGTTAAAT
ISSen13	CP037875	CTT	AGTCGGTATAA	GATGGA	TTATGTTAAAT
ISSen13	KF362121	CTT	AGTGCGTATAA	GATTGA	TTATGTTAAAT
ISSen13	CP058810	CTT	AGTGCGTATAA	GACACA	TTATGTTAAAT
ISKpn65	LT994840	ATACA	GGTGCGCATAA	CAAAGA	TTATGTTAAAT
ISKpn65	CP001630	GAGAT	CTGTAAAGGCT	CAATGC	TTATGTCTAGT
ISKpn65	MK552108	AAGTT	TTGTAACAGCA	AAGTTG	TTATGTCCGCT
ISLad2	CP021463	GATG	AGTGCGCATAA	TCAGGA	TTATGTAAGAT
ISLad2	CP083321	CTTA	AGTGCGCATAA	GCTGGA	TTATGTAAGAT
ISLad2	KX710093	AGTA	AGTTGCTATAA	CGATAC	TTATGGAAAAT
ISBcen27	CP021069	ATCAA	AATGTCGATAA	TGTTGA	TTATGTCAAAT
ISBcen27	FR898703	ACAAA	AATGTTGATAA	TGTTGA	TTATGTCAAAT
ISBcen27	CP000960	ACAAA	AATATCGATAA	TCCGCA	TTATGTCAATC
ISRel10	CP001076	ACACC	AAATAGCATAA	TGCAAG	TTATGGAACTT
ISRel10	CP012125	ACACC	AAATAGCATAA	TCAAAG	TTATGGAACTT
ISRel10	CP020950	ACACC	AAATAGCATAA	TCAACG	TTATGGAACTT
ISMex6	CP001511	GTCGT	CGTTCGCATAA	GCGGTG	TTTTGA CAAGA
ISMex6	CP001510	AAAGC	CGTTCGCATAA	GATATA	TTATGGAACGT
ISMex6	CP029173	CGAGC	TGTTCGCATAA	GATATC	TTATGGAACCT
ISRel26	CP013513	GTAAA	ATGTGGCGTAA	GACGCA	TTATGGAACAA
ISRel26	CP092434	ATAAC	ATATCGCATAA	TCTAAT	TTATGGAACCG
ISRel26	CP000134	ATAAC	ATGTCGCATAA	TCCAAT	TTATGGAACCG

*Red lettering indicates bases that differ from the p*dif* consensus sequence.

ISLad2 was similar, found 4 bp away from a *dif*-like site showing moderate conservation in the XerD site sequence but significant divergence in the XerC site sequence. The other four IS in this group (ISBcen27, ISRel10, ISRel26 and ISMex6) were found 5 bp away from sequences that only very loosely resembled p*dif* sites (Table 8). Interestingly, in all but one case the first five bases of the XerD site were conserved and the four bases closest to the spacer of the potential C site are also conserved in most. This suggests these may be ancient *dif*-like sites that have degenerated, or they may be related to *dif* sites with a different consensus found in these species. In all instances, the targeted sites also appeared to be a single *dif*-like site, rather than a pair of inversely oriented p*dif* sites in a *dif* module configuration.

When the IS with either longer TSD or no detectable TSD were examined in the same way, we found no evidence of the IS targeting any sequence resembling a p*dif* site or any other conserved sequence. In several instances only single examples of the IS were found, or the same structure that been sequenced multiple times. The lack of independent data makes it difficult to determine if they are targeting a different conserved sequence. As more genomes are sequenced additional evidence may emerge.

## Discussion

Here, we have proposed the formation of a unique new IS family made up of a group of related IS currently found in the NCY category in ISFinder. Members of the IS*1202* family all encode a large DDE transposase related to the Tn*7* transposase and share strong similarity in the regions around the conserved acidic residues. The similarities extend to the features of the TIRs and to the presence of additional transposase binding sites near the ends. To date, no other IS family whose members encode a DDE transposase has features related to those of Tn*7* and its closest relatives, making this family unique.

The analysis presented here arose from our earlier observation that a particular group of IS found in plasmids isolated from *Acinetobacter* species appeared to be specifically associated with p*dif* sites [8], suggesting targeting. Here, seven related IS found exclusively in *Acinetobacter* species were always found in a specific orientation 5 bp from the XerC binding site of the *dif* site in the terminus region of the chromosome or a *dif*-like site elsewhere in the chromosome or a p*dif* site in a plasmid that includes *dif* modules. Hence, these IS are *dif* site hunters. Other more distantly related IS listed in ISFinder in the IS*1202* group that also generate a short TSD of 3–5 bp may also target *dif*-like sites, though in some cases the site has clearly diverged or degraded. An IS or Tn targeting the same specific site is relative uncommon. Transposon Tn*7* is the best studied example [21]. However, Tn*7* transposition requires three proteins, the DDE transposase TnsB plus TnsA and TnsC, and a fourth protein TnsD is required for targeted transposition [20, 21]. Here, no additional proteins are present and the features determining target specificity probably reside in the transposase. One possibility is that the ISAjo2 group transposases interact directly with the XerC protein bound to the *dif* or *dif*-like site.

The IS*1202* family includes a second large group of IS, the IS*1202* group, that generate an unusually long TSD and, in the phylogeny of the transposases, form a clearly distinguishable group (Table 7). A third small group do not appear to generate a TSD. Based on available information, members of these groups did not appear to target one or more specific sites. Hence, it may be useful in future to divide the family into two, namely the IS*1202* family and the ISAjo2 family, or even three families.

## Supplementary Data

Supplementary material 1
