# Supplementary material for: Insertion sequences related to ISAjo2 target pdif and dif sites and belong to a new IS family, the IS1202 family
Source: Microb Genom. 2023 Mar 7;9(3):mgen000953. doi: 10.1099/mgen.0.000953 (PMC10132070; doi:10.1099/mgen.0.000953)
Supplement: Supplementary material 1 [file mgen-9-953-s001.pdf]

Table S1. Amino acid identity between the transposases of ISAjo2 group IS

	ISAjo2	ISAba71	ISAso2	ISApi2	ISAba32	ISAba54	ISAlw22
ISAjo2	100	97.05	95.69	92.97	71.3	71.07	71.53
ISAba71		100	95.62	92.34	72.01	71.3	72.23
ISAso2			100	92.12	70.88	70.84	71.11
ISApi2				100	70.2	69.25	70.43
ISAba32					100	93.41	97.07
ISAba54						100	94.32
ISAlw22							100

Cluster 1

```
ISAjo2_L     TGTAACTCCAAAATAGAAATGTCCGGTTTCTCCAAAGTAAAAATGTCCGCTTTTAGAATA
ISAjo2_R     TGTTATTCCTAAGTAGAAATGTCCTACCTAATAACCAAATAGAAATGTCCTATATGGACA
ISAba71_L    TGTAACTCCAAAATAGAAATGTCCGGTTTCTCCAAAGTAAAAATGTCCGCTTTTAGAATA
ISAba71_R    TGTTATTCCCAAGTAGAAATGTCCTACCTAATAACCAAATAGAAATGTCCTATATGGACA
ISApi2_L     TGTAAGCCCAAAATAGAAATGTCCGGTTTCTCCAAAGTAAAAATGTCCGCTTTTAGAATA
ISApi2_R     TGTTATTCCCAAGTAGAAATGTCCTACCTAATAACCAAATAGAAATGTCCTATATGGACA
ISAso2_L     TGTAAGCCCCAAGTAGAAATGTCCGGTTTCTCCAAAGTAAAAATGTCCGCTTTTAGAATA
ISAso2_R     TGTTATTCCTAAGTAGAAATGTCCTACCTAATAACCAAATAGAAATGTCCTATACAGACA
```

TIR

Cluster 2

```
ISAba32_L    TGTTAGAACCCATTTAAAGTGTCTATATTCTCACCAATAAAAATGTCTGGTTTATGATGG
ISAba32_R    TGTTATTCACCAATTAAAATGTCTATGCGAAAAACCATTTAAAATGTCCTGTTTTTAACC
ISAlw22_L    TGTTAGAACCCATTTAAAGTGTCTATATTCTCACCAATAAAAATGTCTGGTTTATGATGG
ISAlw22_R    TGTTATTCACCAATTAAAATGTCTATGCGATAAACCATTTAAAATGTCCTGTTTTTAACC
ISAba54_L    TGTTAGAAACCATTTAAAGTGTCTGTATTCTCACCAATAAAAATGTCTGGTCTATGATGC
ISAba54_R    TGTTATTCACCAATTAAAATGTCTATGCGATAAACCATTTAAAATGTCCTGTTTTTAACC
```

TIR

Fig. S1. Terminal inverted repeats of ISAjo2-type IS. Repeated sequences are denoted by red text. The 24 bp terminal inverted repeat is marked below by a black bar.

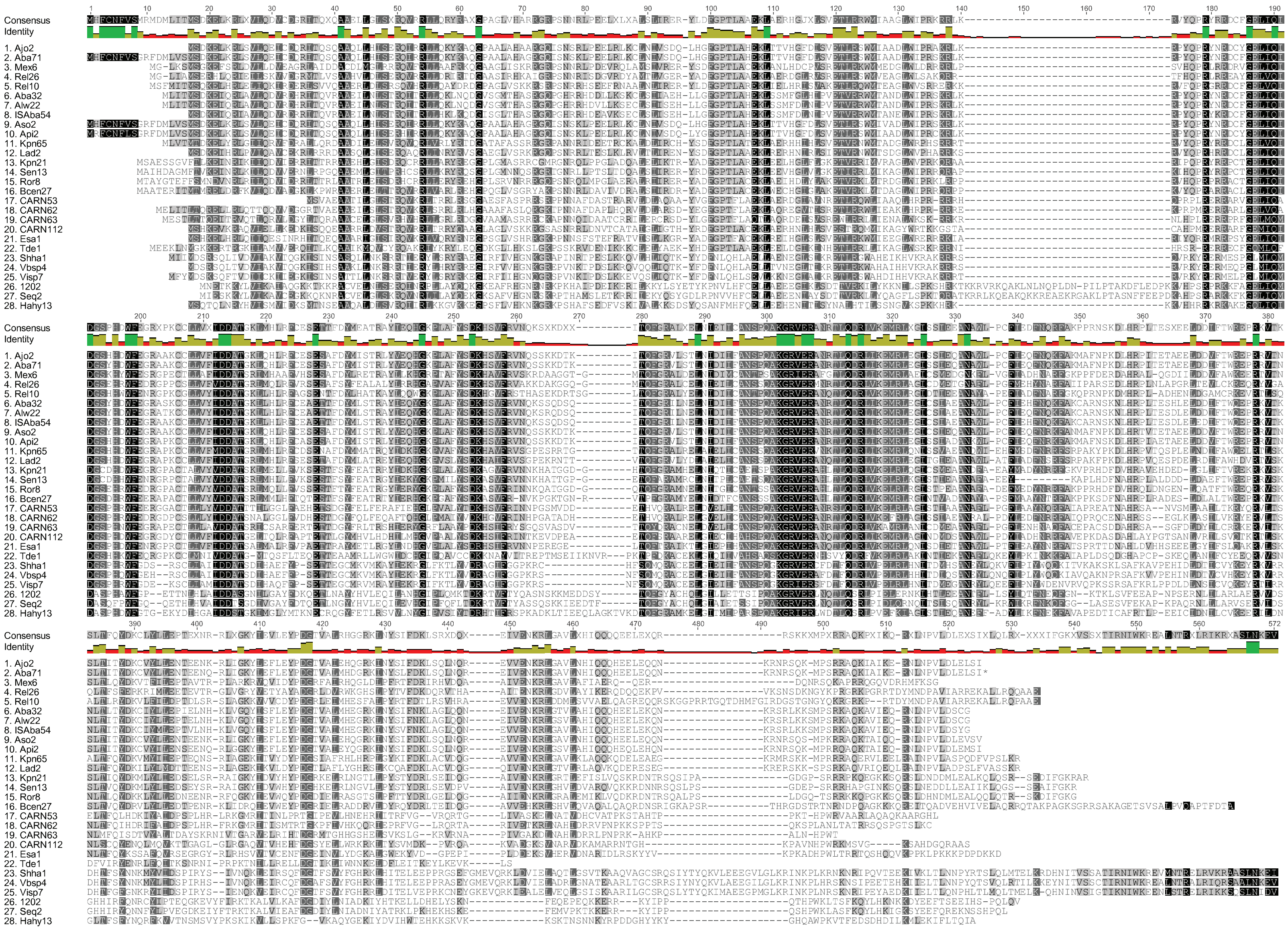

Consensus
Identity
1. Ajo2
2. Aba71
3. Mex6
4. Rel26
5. Rel10
6. Aba32
7. Alw22
8. ISAba54
9. Aso2
10. Api2
11. Kpn65
12. Lad2
13. Kpn21
14. Sen13
15. Ror8
16. Bcen27
17. CARN53
18. CARN62
19. CARN63
20. CARN112
21. Esa1
22. Tde1
23. Shha1
24. Vbsp4
25. Visp7
26. 1202
27. Seq2
28. Hahy13

Fig. S2. Alignment of the amino acid sequences of the IS*1202* family transposases. Amino acids are shaded as follows: black, 100% of aa are similar; dark grey, 80-99% similarity; light grey, 60-79% similarity; unshaded, less than 59% similarity.
